# 38766 Massively Parallel Reporter Assay Reveals Functional Impact of 3™-UTR SNPs Associated with Neurological and Psychiatric Disorders

**DOI:** 10.1017/cts.2021.645

**Published:** 2021-03-31

**Authors:** Andy B. Chen, Kriti Thapa, Hongyu Gao, Jill L. Reiter, Junjie Zhang, Xiaoling Xuei, Hongmei Gu, Yue Wang, Howard J. Edenberg, Yunlong Liu

**Affiliations:** Indiana University School of Medicine

## Abstract

ABSTRACT IMPACT: Screening the effect of thousands of non-coding genetic variants will help identify variants important in the etiology of diseases OBJECTIVES/GOALS: Massively parallel reporter assays (MPRAs) can experimentally evaluate the impact of genetic variants on gene expression. In this study, our objective was to systematically evaluate the functional activity of 3’-UTR SNPs associated with neurological disorders and use those results to help understand their contributions to disease etiology. METHODS/STUDY POPULATION: To choose variants to evaluate with the MPRA, we first gathered SNPs from the GWAS Catalog that were associated with any neurological disorder trait with p-value < 10-5. For each SNP, we identified the region that was in linkage disequilibrium (r2 > 0.8) and retrieved all the common 3’-UTR SNPs (allele-frequency > 0.05) within that region. We used an MPRA to measure the impact of these 3’-UTR variants in SH-SY5Y neuroblastoma cells and a microglial cell line. These results were then used to train a deep-learning model to predict the impact of variants and identify features that contribute to the predictions. RESULTS/ANTICIPATED RESULTS: Of the 13,515 3’-UTR SNPs tested, 400 and 657 significantly impacted gene expression in SH-SY5Y and microglia, respectively. Of the 84 SNPs significantly impacted in both cells, the direction of impact was the same in 81. The direction of eQTL in GTEx tissues agreed with the assay SNP effect in SH-SY5Y cells but not microglial cells. The deep-learning model predicted sequence activity level correlated with the experimental activity level (Spearman’s corr = 0.45). The deep-learning model identified several predictive motifs similar to motifs of RNA-binding proteins. DISCUSSION/SIGNIFICANCE OF FINDINGS: This study demonstrates that MPRAs can be used to evaluate the effect of non-coding variants, and the results can be used to train a machine learning model and interpret its predictions. Together, these can help identify causal variants and further understand the etiology of diseases.

